# The health and well-being of men who have sex with men (MSM) in Britain: Evidence from the third National Survey of Sexual Attitudes and Lifestyles (Natsal-3)

**DOI:** 10.1186/s12889-016-3149-z

**Published:** 2016-07-07

**Authors:** Catherine H. Mercer, Philip Prah, Nigel Field, Clare Tanton, Wendy Macdowall, Soazig Clifton, Gwenda Hughes, Anthony Nardone, Kaye Wellings, Anne M. Johnson, Pam Sonnenberg

**Affiliations:** Research Department of Infection and Population Health, University College London, 3rd floor Mortimer Market Centre, London, UK; Department of Social and Environmental Health Research, London School of Hygiene and Tropical Medicine, Tavistock Place, London, UK; Public Health England, Colindale, London, UK

## Abstract

**Background:**

To date, research on men who have sex with men (MSM) has largely focused on their sexual health needs and on men recruited from gay-orientated venues. National probability survey data provide a rare opportunity to examine the broader sociodemographic, behavioural, and health profiles of MSM, defined as men who reported ≥1 male sexual partner(s) in the past 5 years, and thus regardless of their sexual identity, in comparison to men reporting sex exclusively with women (MSEW) during this time, and also the extent that health inequalities cluster.

**Methods:**

Britain’s third National Survey of Sexual Attitudes and Lifestyles (Natsal-3), a probability sample survey, interviewed 15,162 people aged 16–74 years (6,293 men) during 2010–2012 using computer-assisted personal-interviewing with a computer-assisted self-interview. We used multivariable regression to compare MSM relative to MSEW in their reporting of variables, individually and collectively, corresponding to three domains: physical, mental, and sexual health.

**Results:**

Among all men, 2.6 % (n = 190) were defined as MSM, of whom 52.5 % (95 % CI: 43.6 %–61.2 %) identified as gay. MSM were as likely as MSEW (n = 5,069) to perceive their health was ‘bad’/’very bad’, despite MSM being more likely to report a long-standing illness/disability/infirmity (adjusted odds ratio, AOR: 1.46, 95 % CI:1.02–2.09), treatment for depression/past year (2.75, 1.69–4.47), and substance use (e.g., recreational drug use/past year: 3.46, 2.22–5.40). MSM were more likely to report harmful sexual health behaviours, e.g., condomless sex with ≥2 partners/past year (3.52, 2.13–5.83), and poor sexual health outcomes, including STI diagnosis/es (5.67, 2.67–12.04), poorer sexual function (2.28, 1.57–3.33), both past year, and ever-experience of attempted non-volitional sex (6.51, 4.22–10.06). MSM were also more likely than MSEW to report poor health behaviours and outcomes both within and across the three health domains considered. Of all MSM, 8.4 % had experienced poor health outcomes in all three domains – physical, mental, and sexual health - in contrast to 1.5 % of all MSEW.

**Conclusions:**

MSM are disproportionately affected by a broad range of harmful health behaviours and poor health outcomes. Although often observed for a minority of MSM, many health inequalities were seen in combination such that policies and practices aimed at improving the health and well-being of MSM require a holistic approach, regardless of clinical specialty.

## Background

Men who have sex with men (MSM) bear a disproportionate burden of sexually transmitted infections (STIs), including HIV [1, 2]. However, sexual health extends beyond STI/HIV, and includes sexual function and sexual violence [3]. Furthermore, good sexual health is increasingly recognised as a key component of general health and well-being [4], with health behaviours and outcomes often sharing common risk factors that can exacerbate one another [5]. Of note, data suggest that gay men are at increased risk of poor mental health [6] and substance use [7], which can increase their likelihood of experiencing poor sexual health outcomes, including STI/HIV transmission [5].

To date, much of the evidence regarding MSM is based on those who attend sexual health clinics [8], or convenience surveys targeting MSM identifying as gay and/or recruit from gay-orientated venues [9], neither of which are representative of the diverse population of MSM [10]. In contrast, Britain’s National Surveys of Sexual Attitudes and Lifestyles (Natsal) use probability sampling so the data can be considered as broadly representative of the general population [11], and include MSM regardless of their sexual identity. In this paper we use data from the most recent Natsal, Natsal-3, to examine the sociodemographic profile and sexual, mental and physical health of MSM, comparing them to men who reported sex exclusively with women (MSEW). We explore the extent that harmful health behaviours and poor health outcomes are reported in combination, and thus cluster within and across health domains, to capture the extent that inequalities are experienced by MSM relative to MSEW.

## Methods

In Natsal-3, households across Britain were selected using stratified probability sampling from which one eligible individual was selected at random and invited to participate. Unlike Natsal-2 [12], there was no geographical over-sampling, for example, of areas that have large gay populations [13]. Altogether, 15,162 men and women aged 16–74 years (6,293 men) were interviewed. The overall response rate was 57.7 % (of all addresses known or estimated to be eligible) consistent with other major social surveys undertaken contemporaneously, and the co-operation rate was 65.8 % (of all contacted addresses known to be eligible). Full details of the methodology have been published [11].

Participants completed a combination of a face-to-face computer-administered personal interview (CAPI) and a computer-assisted self-interview (CASI). Participants were routed into the CASI if they reported sex with an opposite-sex partner and/or reported any sexual experience with someone of the same-sex (both since age 13), or if they refused to answer this question. The CASI included many questions about sexual behaviour, including: *“Have you had sex with a man involving genital contact? That is oral or anal sex or any other contact involving the genital area*.” Men who reported that they had were then asked with how many men they had had sex with in various timeframes. In this paper we define MSM as men who reported sex with at least one man in the five years prior to interview. Participants were asked similar questions about their opposite-sex partners which we used to identify men who reported sex exclusively with women (MSEW) in the same timeframe, the five years prior to interview.

The CASI asked about participants’ sexual health including their experience of STI diagnosis and non-volitional sex. We assessed sexual function with the Natsal-SF, a validated measure that considers problems with sexual response, sexual function in the relationship context, and self-appraisal of sex life, among those sexually-active (defined as reporting sex in the past year) [14]. The CASI also asked about use of sexual health services including HIV testing, sexual health clinic attendance, and whether participants had sought help/advice about their sex life and if so, from where/whom.

Most of the data on general health and well-being were collected face-to-face using showcards so that participants needed only to report a letter code. These included questions about alcohol consumption, and experience of a range of chronic conditions, considered here as reporting none, one, or more than one such condition to capture co-morbidity [4]. Reporting receiving treatment for depression from a health professional in the past year was one such condition but we considered it separately in order to consider mental health as a separate domain. Participants were deemed to have had depressive symptoms in the past two weeks if they scored a total of at least three in response to the validated two-question Patient Health Questionnaire (PHQ-2) [15, 16], which was included in the CASI.

After the CASI, questions about STI/HIV risk perception were asked face-to-face using showcards with Likert scale response options, followed by standard demographic items regarding ethnicity, sexual identity, social class (as measured by the National Statistics Socio-Economic Classification [17]), and area-level deprivation (as measured by the Index of Multiple Deprivation [18]).

The Natsal-3 study was approved by the Oxfordshire Research Ethics Committee A (reference: 09/H0604/27). Participants provided oral informed consent for interviews.

### Statistical analysis

We did all analyses using the complex survey functions of Stata (version 13) to incorporate the weighting, clustering, and stratification of Natsal-3. We calculated descriptive statistics to compare MSM with MSEW, presenting age-standardised estimates to account for differences in age distributions. We used ordinal and binary multivariable logistic regression to calculate adjusted odds ratios (AORs) to investigate how the reporting of harmful health behaviours and poor health outcomes differed for MSM relative to MSEW.

We then considered the extent that MSM and MSEW reported multiple harmful behaviours *within* two health domains: physical and sexual health by summing the number of specific behaviours reported (a total of four harmful physical behaviours and three harmful sexual behaviours (Table 1)). We then used proportional Venn diagrams to consider the extent of overlap *across* health domains as the proportion reporting harmful behaviours in both health domains. We used the same approach to examine the reporting of multiple poor health outcomes within and across three different health domains: physical, sexual, and mental health (Table 1).

**Table 1:** Harmful behaviours and poor health outcomes reported in Natsal-3 used to define health domains

Harmful behaviours	Poor health outcomes
Domain: Mental health	
*Not available in Natsal-3*	• Received treatment for depression from a health professional, past year
Domain: Physical health	
• Current smoker • Usual alcohol consumption greater than recommended levels of 21 units per week [34] • Usually drink >8 units of alcohol on one occasion (‘binge drinking’ [34]) at least weekly • Any illicit drug use (including cannabis), past year	• Current health described as ‘bad’ or ‘very bad’ • Long-standing illness/disability/infirmity • 1+ chronic condition (excluding depression) • Unhealthy BMI (BMI <18 | >25) [35]
Domain: Sexual health	
• ≥2 condomless-sex partners, past year • ≥10 sex partners, past year • Non-use of condoms at first sex with any of the three most recent partners, past year	• STI diagnosis/es, past year • Distressed/worried about sex life, past year • Experienced attempted non-volitional sex, ever

While Natsal-3 collected data on sexual identity, we do not present estimates for MSM stratified according to their sexual identity due to a lack of statistical power to make meaningful comparisons, but for reference our tables include estimates for the subset of MSM who identified as gay, including adjusted odds ratios relative to MSEW. We then summarise the differences observed relative to comparing all MSM to MSEW at the end of the Results section.

We used an α of 0.05 in all analyses.

## Results

### Prevalence of MSM & reported numbers of sexual partners

Among men aged 16–74 years resident in Britain, 2.6 % [95 % CI: 2.1 %–3.0 %] were defined as MSM, corresponding to an unweighted total of 190 MSM in Natsal-3. Of these men, 107 [52.5 %, 95 % CI: 43.6 %–61.2 %] identified as gay, 34 [18.6 %, 95 % CI: 12.8 %–26.3 %] as bisexual, and 45 [28.4 %, 95 % CI: 20.8 %–37.5 %] as heterosexual/straight.

MSM reported larger numbers of sexual partners - of either gender - than MSEW in all timeframes studied (Table 2). In the five years prior to interview, the majority of MSEW (62.7 %) reported one partner only, while this number was reported by 15.6 % of MSM, with 34.8 % of MSM reporting at least ten partners during this time, as did 6.3 % of MSEW.

**Table 2:** Sexual partner numbers in the lifetime, past 5 years, and past year reported by men who have sex exclusively with women (MSEW), all men who have sex with men (MSM), and MSM who identified as gay in Natsal-3

	All sexual partners (regardless of gender), reported by:			Male sexual partners, reported by:			Female sexual partners, reported by:		
	MSEW	All MSM	MSM who identified as gay^b^	MSEW	All MSM	MSM who identified as gay^b^	MSEW	All MSM	MSM who identified as gay^b^
*Denominators* ^*a*^ *:*	*5069, 6257*	*190, 191*	*107, 99*	*5069, 6257*	*190, 191*	*107, 99*	*5069, 6257*	*190, 191*	*107, 99*
Timeframe:									
Lifetime									
Age standardised:									
% [95 % CI]									
0	0	0	0	97.5 [97.0–98.0]	0	0	0	30.1 [23–38.3]	55.4 [42.7–67.4]
1	13.7 [12.6–14.9]	1.8 [0.7–4.5]	1.3 [0.3–5.3]	1.4 [1.1–1.9]	14.6 [9.3–22.2]	3.1 [0.8–10.7]	13.7 [12.6–14.9]	13.7 [9.0–20.3]	22.1 [13.6–33.8]
2	8.4 [7.5–9.3]	1.8 [0.9–3.8]	1.5 [0.5–4.9]	0.5 [0.4–0.8]	9.3 [5.2–16.0]	4.4 [1.8–10.4]	8.4 [7.5–9.4]	8.5 [4.6–15.0]	8.8 [3.5–20.4]
3–4	15.3 [14.1–16.5]	9.2 [5.2–15.9]	8 [3.8–16.3]	0.3 [0.1–0.5]	15.1 [9.8–22.3]	10 [5.1–18.8]	15.6 [14.4–16.8]	9.6 [5.7–15.9]	6.2 [2.3–15.3]
5–9	25.7 [24.3–27.1]	19.1 [13.3–26.6]	18.1 [11.3–27.5]	0.2 [0.1–0.4]	18.5 [12.3–27]	12.9 [7.6–21.0]	25.4 [24–26.8]	11.3 [6.6–18.7]	4.8 [1.8–11.9]
10+	37 [35.5–38.6]	68 [59.5–75.4]	71.1 [61.3–79.3]	0.1 [0–0.3]	42.5 [34.3–51.2]	69.6 [59.9–77.9]	37 [35.5–38.5]	26.9 [19.5–35.8]	2.8 [1.1–6.8]
Mean	14.3	111.1	86.7	0.1	45.7	84.8	14.3	60.4	1.2
aAOR^c^	1.00	3.40 (2.42–4.78)	3.42 (2.25–5.22)	1.00	836 (516–1356)	2504 (1191–5267)	1.00	0.15 (0.07–0.32)	0.00 (0.00–0.01)
Non-age standardised:									
Mean (sd)	14.3 (43.1)	90.1 (345.0)	76.4 (166.6)	0.1 (0.5)	44.7 (121.6)	74.2 (165.5)	14.3 (43.1)	44.6 (323.6)	1.3 (3.2)
Median (IQR)	6 (3–15)	17 (6–50)	19 (6–70)	0 (0–0)	6 (2–25)	19 (5–70)	6 (3–5)	2 (0–9)	0 (0–1)
Past 5 years									
Age standardised:									
% [95 % CI]									
0	0	0	0	N/A			0	54.1 [45.7–62.3]	93.5 [89.6–96.1]
1	62.7 [61.3–64.0]	15.6 [10.1–23.3]	26.7 [16.5–40.3]		32.2 [24.2–41.5]	26.3 [16.2–39.8]	62.7 [61.3–64]	16.1 [10.5–23.9]	3.3 [1.6–6.5]
2	11.4 [10.5–12.4]	10.7 [6.4–17.5]	5.5 [2.5–11.9]		13.5 [8.5–20.8]	6.8 [3.4–13.0]	11.4 [10.5–12.4]	5.5 [2.5–11.3]	1.4 [0.4–4.7]
3–4	11.3 [10.4–12.2]	15.3 [10–22.8]	19.4 [11–31.7]		15.7 [10.4–23.1]	19.1 [10.9–31.3]	11.3 [10.4–12.2]	9.7 [5.3–16.9]	1.4 [0.4–4.7]
5–9	8.4 [7.7–9.2]	22.6 [16.4–30.3]	13.5 [7.1–24.3]		11.9 [7.5–18.3]	12.6 [6.4–23.3]	8.4 [7.7–9.2]	6.1 [3.1–11.7]	0.4 [0–2.5]
10+	6.3 [5.7–6.9]	35.8 [27.8–44.8]	34.8 [23.7–47.9]		26.7 [19.7–35.2]	35.3 [24.2–48.1]	6.3 [5.7–6.9]	8.5 [4.7–15.2]	0
Mean	3	15.8	17.3		12.2	17.5	3.0	3.7	0.1
aAOR^c^	1.00	9.36 (6.23–14.08)	6.22 (3.57–10.84)				1.00	0.01 (0.00–0.03)	0.00 (0.00–0.00)
Non-age standardised:									
Mean (sd)	3.0 (5.3)	15.9 (35.2)	18.1 (43.5)		12.3 (31.7)	18.3 (43.7)	3.0 (5.3)	3.6 (16.6)	0.2 (0.7)
Median (IQR)	1 (1–3)	6 (2–16)	5 (2–30)		3 (1–10)	5 (2–30)	1 (1–3)	0 (0–2)	0 (0–0)
Past year				N/A					
Age standardised:									
% [95 % CI]									
0	6.1 [5.4–6.9]	3.9 [1.9–8.1]	6 [2.2–15.5]		14.9 [9.6–22.2]	5.9 [2.2–15.3]	6.1 [5.4–6.9]	60.8 [51.8–69.1]	97.7 [94.1–99.2]
1	77.1 [75.8–78.4]	36.0 [27.8–45.1]	45.8 [33.2–59.0]		42.6 [33.9–51.7]	45.3 [32.8–58.4]	77.1 [75.8–78.4]	21.6 [15.0–30.2]	0.8 [0.1–5.1]
2	8.0 [7.2–8.8]	13 [8.0–20.4]	8.5 [3.9–17.6]		9.7 [5.8–15.7]	9.0 [4.3–17.9]	8.0 [7.2–8.8]	6.2 [3.2–11.8]	0.7 [0.2–2.9]
3–4	5.4 [4.8–6.1]	16.1 [10.8–23.5]	12.8 [7–22.4]		13.1 [8.6–19.6]	13.9 [7.7–23.8]	5.4 [4.8–6.1]	7.1 [3.7–13.0]	0.7 [0.1–5.1]
5–9	2.2 [1.9–2.7]	17.6 [11.7–25.5]	7.3 [2.8–17.4]		8.6 [4.7–15.3]	6.4 [2.2–16.9]	2.2 [1.9–2.7]	3.7 [1.5–9.0]	0
10+	1.2 [0.9–1.5]	13.4 [8.0–21.7]	19.6 [10.4–34.0]		11.2 [6.4–18.8]	19.5 [10.4–33.7]	1.2 [0.9–1.5]	0.7 [0.1–4.5]	0
Mean	1.4	4.5	5.0		3.5	4.9	1.4	0.9	0
aAOR^c^	1.00	8.29 (5.25–13.09)	4.84 (2.50–9.37)				1.00	0.04 (0.02–0.08)	0.00 (0.00–0.00)
Non-age standardised:									
Mean (sd)	1.4 (2.0)	4.5 (8.5)	5.1 (11.7)		3.5 (8.4)	4.9 (11.7)	1.5 (2.0)	0.9 (1.8)	0.1 (0.4)
Median (IQR)	1 (1–1)	2 (1–5)	1 (1–4)		1 (1–4)	1 (1–4)	1 (1–1)	0 (0–1)	0 (0–0)
New partner, past year				N/A					
Age standardised:									
% [95 % CI]									
0	77.7 [76.6–78.8]	43.3 [34.8–52.2]	49.8 [37.1–62.5]		55.7 [47.0–64.1]	50.8 [38.1–63.4]	77.7 [76.6–78.8]	80.1 [72.4–86.1]	97.7 [94.1–99.2]
1	13.2 [12.3–14.2]	14.8 [10.1–21.3]	15.6 [9.3–25.1]		13.1 [9.1–18.6]	15.6 [9.3–25.0]	13.2 [12.3–14.2]	9.6 [5.6–16.0]	1.9 [0.6–5.7]
2	4.5 [4.0–5.1]	7.8 [4.1–14.2]	4.5 [1.7–11.6]		6.1 [3.3–11.0]	4.8 [1.9–11.8]	4.5 [4.0–5.1]	5.2 [2.7–9.9]	0.4 [0.0–2.5]
3–4	2.7 [2.3–3.1]	12.3 [7.9–18.8]	9.5 [4.3–19.5]		9.7 [6.0–15.4]	9.3 [4.1–19.8]	2.7 [2.3–3.1]	2.7 [0.9–8.1]	0
5–9	1.4 [1.1–1.7]	15.6 [9.5–24.5]	14.1 [6.1–29.2]		10.0 [5.0–18.8]	13.1 [5.4–28.4]	1.4 [1.1–1.7]	1.7 [0.5–6.0]	0
10+	0.5 [0.4–0.7]	6.2 [3.2–11.5]	6.5 [2.9–13.9]		5.4 [2.7–10.5]	6.4 [2.9–13.6]	0.5 [0.4–0.7]	0.7 [0.1–4.5]	0
Mean	0.5	3.2	3.6		2.6	3.5	0.5	0.5	0
aAOR^c^	1.00	6.24 (3.86–10.08)	4.32 (2.35–7.95)				1.00	0.76 (0.47–1.23)	0.08 (0.03–0.22)
Non-age standardised:									
Mean (sd)	0.5 (1.4)	3.2 (8.5)	3.9 (11.8)		2.6 (8.3)	3.7 (11.8)	0.5 (1.4)	0.5 (1.6)	0.0 (0.2)
Median (IQR)	0 (0–0)	1 (0–4)	1 (0–3)			1 (0–3)	0 (0–0)	0 (0–0)	0 (0–0)
Number of condomless-sex partners, past year				N/A					
Age standardised:									
% [95 % CI]									
0	19.5 [18.3–20.9]	43.1 [34.4–52.3]	43.4 [32.3–55.3]		42.9 [32.6–54.0]	30.4 [20.0–43.3]	19.3 [18.1–20.6]	73.7 [65.5–80.5]	98.8 [95.0–99.7]
1	72.4 [70.9–73.8]	32.2 [24.5–41.0]	40.1 [29.2–52.1]		37.1 [27.4–48.1]	49.7 [36.6–62.9]	72.7 [71.3–74.1]	14.7 [9.5–22.1]	1.2 [0.3–5.0]
2+	8.0 [7.3–8.9]	24.7 [17.6–33.4]	16.4 [8.8–28.8]		19.9 [11.7–31.9]	19.8 [10.4–34.6]	7.9 [7.2–8.8]	11.6 [7.3–17.9]	0
Mean	1.0	1.2	1.0		1.1	1.3	1.0	0.5	0.01
aAOR^c^	1.00	0.60 (0.30–1.18)	0.43 (0.21–0.88)				1.00	0.10 (0.06–0.16)	0.00 (0.00–0.01)
Non-age standardised:									
Mean (sd)	1.0 (1.0)	1.2 (2.6)	1.2 (3.1)		1.2 (2.7)	1.5 (3.2)	1.0 (1.0)	0.5 (1.4)	0.01 (0.1)
Median (IQR)	1 (1–1)	1 (0–1)	1 (0–1)		1 (0–1)	1 (0–1)	1 (1–1)	0 (0–1)	0 (0–0)

^a^Unweighted, weighted denominators defined as participants who reported at least one sexual partner in the 5 years prior to interview

^b^MSM who identified as gay in Natsal-3 are a subset of all MSM

^c^Age-adjusted odds ratio (aAOR) for comparing to MSEW

Most MSM (69.9 %) reported at least one *female* partner in their lifetime, with a median of 2 female partners in contrast to a median of 6 among MSEW. Female partners accounted for 22.9 % of all partners reported by MSM. Among MSEW, 2.5 % reported at least one *male* partner to date but, by definition, not in the past five years. Similar proportions of MSM and MSEW - one in five - reported at least one new female partner in the past year.

### Sociodemographic profiles

MSM were slightly younger than MSEW (Table 3). Around 40 % of MSM were neither living with a partner nor in a steady relationship in contrast to 17.8 % of MSEW. MSM were more likely than MSEW to describe their ethnicity as white (96.8 % vs. 88.4 %), and less likely to consider religion as fairly/very important (23.4 % vs. 33.8 %). MSM had higher educational attainment than MSEW, with some evidence of higher socio-economic status, but no difference in area-level deprivation.

**Table 3:** Sociodemographic profile of men who have sex exclusively with women (MSEW), all men who have sex with men (MSM), and MSM who identified as gay in Natsal-3

	MSEW	All MSM		MSM who identified as gay^b^	
*Denominators* ^*a*^ *:*	*5069, 6257*	*190, 191*		*107, 99*	
	% [95 % CI]	% [95 % CI]	*p*-value^*^	% [95 % CI]	*p*-value^**^
Age group					
16–34	35.0 [33.6–36.5]	43.6 [35.9–51.6]	0.106	51.3 [40.2–62.3]	<0.001
35–54	41.0 [39.3–42.8]	38.3 [30.3–47.0]		41.2 [30.2–53.1]	
55–74	23.9 [22.5–25.4]	18.1 [12.1–26.2]		7.5 [3.7–14.8]	
Median (IQR)	41 (30–54)	40 (26–51)		34 (26–46)	
	*Age-standardised estimates:*				
Relationship status					
Married/civil partnership	54.4 [53.1–55.7]	25.6 [18.2–34.8]	<0.001	14.9 [8.0–26.0]	<0.001
Living with partner	16.0 [14.9–17.1]	20.6 [14.3–28.8]		21.8 [12.8–34.7]	
In a ‘steady’ relationship but not living together	11.8 [11.0–12.7]	14.5 [9.9–20.7]		15.8 [9.5–25.0]	
Not in a ‘steady’ relationship	17.8 [16.9–18.8]	39.3 [31.5–47.6]		47.6 [35.5–59.9]	
Ethnicity - white	88.4 [87.2–89.5]	96.8 [91.6–98.8]	0.007	98.9 [92.9–99.8]	0.013
Religion/religious beliefs –fairly/very important	33.8 [32.3–35.4]	23.4 [16.9–31.4]	0.007	27.3 [17.6–39.9]	0.056
Academic qualifications^c^					
No academic qualifications	17.9 [16.7–19.2]	10.2 [6.0–16.9]	0.017	9.3 [4.2–19.5]	0.010
Academic qualifications typically gained at age 16	34.3 [32.8–35.8]	33.8 [26.2–42.2]		31.7 [21.2–44.5]	
Studying for/attained further academic qualifications	47.8 [46.1–49.5]	56.0 [47–64.6]		58.9 [45.7–71.0]	
National Statistics Socio-Economic Classification^d^					
Managerial & professional occupations	38.1 [36.4–39.7]	43.1 [34.6–52]	0.086	43.8 [31.9–56.5]	0.094
Intermediate occupations	17.1 [15.8–18.4]	20.0 [13.7–28.3]		26.5 [16.4–39.8]	
Semi-routine/routine occupations	32.6 [31.1–34.2]	27.5 [20.1–36.4]		18.9 [11.9–28.8]	
Never worked and long-term unemployed	5.0 [4.4–5.7]	1.2 [0.4–3.9]		3.7 [0.9–13.7]	
Full-time students	7.2 [6.5–8]	8.2 [54–12.3]		7.1 [3.8–12.8]	
Lives in an urban area^e^	76.6 [74.8–78.3]	81.5 [72.9–87.9]	0.118	89.1 [79.9–94.4]	0.005
Quintile of Index of Multiple Deprivation^f^					
1 (least deprived)	21.2 [19.7–22.8]	18.9 [12.7–27.2]	0.530	13.1 [7.5–22.0]	0.116
2	21.6 [20.1–23.2]	16.6 [11.0–24.3]		17.2 [9.2–29.8]	
3	19.4 [17.9–20.9]	21.0 [15.0–28.7]		16.9 [10.1–26.8]	
4	19.6 [18.1–21.2]	21.2 [15.1–29.0]		26.2 [17.3–37.6]	
5 (most deprived)	18.2 [16.8–19.6]	22.2 [16.2–29.6]		26.6 [18.6–36.6]	

^*^ *p*-value for comparing all MSM to MSEW

^**^ *p*-value for comparing MSM who identified as gay in Natsal-3 to MSEW. Note that MSM who identify as gay are a subset of all MSM

^a^Unweighted, weighted denominators defined as participants who reported at least one sexual partner in the 5 years prior to interview

^b^MSM who identified as gay in Natsal-3 are a subset of all MSM

^c^Participants aged ≥17 years

^d^National Statistics Socio-Economic Classification is a measure of individual socio-economic status [17]

^e^Defined as areas with a population of at least 10,000 people

^f^Index of Multiple Deprivation is an area-level measure of deprivation [18]

### Health behaviours and outcomes

Alcohol consumption was similar between MSM and MSEW (Table 4), but MSM were more likely to report being a current smoker (aAOR:1.72), and any recreational drug use (past year, aAOR:3.46), in particular, using drugs other than cannabis, which was reported by one-quarter of all MSM. There was no difference between MSM and MSEW in their reporting of having ever injected non-prescribed drugs. A minority of MSM and MSEW described their health as ‘bad’/‘very bad’. Similarities also existed in the proportions reporting chronic health conditions, but MSM were more likely than MSEW to report that they had a long-standing illness, disability, or infirmity (aAOR:1.46), perceive they had health condition(s)/disability/ies that had affected their sexual activity/enjoyment (past year, aAOR:2.24), and report taking medications they perceived limited their sexual activity/enjoyment (past year, aAOR:2.56). The proportions screening positive for depressive symptoms using the PHQ-2 [15, 16] were similar at around 10 % but MSM were more likely than MSEW to report having received treatment for depression (past year, aAOR:2.75).

**Table 4:** Health behaviours and outcomes reported by men who have sex exclusively with women (MSEW), all men who have sex with men (MSM) and MSM who identified as gay in Natsal-3

	MSEW	All MSM		MSM who identified as gay^b^	
*Denominators:* ^*a*^	*5069, 6257*	*190, 191*		*107, 99*	
	% [95 % CI]	% [95 % CI]	*p*-value^*^	% [95 % CI]	*p*-value^**^
*Health behaviours*					
Usual alcohol consumption more than recommended levels^c^	8.8 [8–9.8]	11.4 [7.2–17.6]		10.3 [5.7–18.0]	
aAOR [95 % CI]^e^	1.00	1.43 [0.87–2.36]	0.156	1.67 [0.87–3.21]	0.125
Usual frequency of binge drinking^d^					
Never or rarely	60.7 [59.1–62.3]	54.0 [44.9–62.9]		58.6 [48–68.5]	
Monthly	17.5 [16.4–18.8]	24.9 [17.3–34.4]		25.4 [16.6–36.8]	
**Weekly or daily**	21.7 [20.4–23.1]	21.1 [15.1–28.7]		16.0 [9.9–24.8]	
aAOR [95 % CI]^e,f^	1.00	0.99 [0.66–1.49]	0.972	0.84 [0.49–1.45]	0.535
Smoking status					
Never	47.2 [45.6–48.9]	29.5 [22.7–37.4]		30.2 [21.5–40.6]	
Ex-smoker	25.9 [24.5–27.4]	32.6 [25.2–41]		38.9 [28.8–49.9]	
**Current smoker**	26.8 [25.4–28.3]	37.9 [30–46.4]		30.9 [22.1–41.4]	
aAOR [95 % CI]^e,f^	1.00	1.72 [1.21–2.45]	0.003	1.37 [0.87–2.16]	0.173
Recreational drug use, past year					
None	84.3 [83.2–85.3]	64.0 [56–71.3]		59.1 [47.4–69.9]	
**Cannabis only**	9.1 [8.3–9.9]	10.2 [6.1–16.7]		8.1 [3.2–19]	
**Cannabis and/or other drugs**	6.7 [6.0–7.4]	25.8 [19.3–33.6]		32.8 [23.9–43.2]	
aAOR [95 % CI]^e,f^	1.00	3.46 [2.22–5.40]	<0.001	4.18 [2.33–7.49]	<0.001
Injecting drug use, ever	1.3 [1.0–1.7]	0.7 [0.2–2.8]		0.8 [0.1–5.4]	
aAOR [95 % CI]^e^	1.00	0.49 [0.11–2.08]	0.332	0.53 [0.07–3.90]	0.529
Taken any medications that have limited your sexual activity or enjoyment, past year	7.8 [7.0–8.7]	16.8 [11.3–24.2]		21.5 [12.8–33.9]	
aAOR [95 % CI]^e^	1.00	2.56 [1.57–4.19]	<0.001	3.24 [1.77–5.94]	<0.001
*Health outcomes*					
Self-reported health status [‘bad’/‘very bad’]^g^	3.1 [2.7–3.7]	5.2 [2.7–9.7]		9.5 [4.1–20.5]	
aAOR [95 % CI]^e^	1.00	1.67 [0.88–3.18]	0.117	2.23 [0.99–5.01]	0.052
≥1 long-standing illness/disability/infirmity	29.9 [28.5–31.4]	38.1 [30.8–45.9]		44.1 [34.7–53.9]	
aAOR [95 % CI]^e^	1.00	1.46 [1.02–2.09]	0.041	1.65 [1.03–2.67]	0.039
Number of chronic conditions^h^					
0	68.7 [67.2–70.1]	66 [57.5–73.6]		58.4 [50.4–65.9]	
**1**	20.6 [19.3–22.0]	20.2 [14.0–28.2]		22.7 [13.6–35.4]	
**≥2**	10.7 [9.8–11.8]	13.8 [8.8–21.0]		18.9 [11.0–30.7]	
aAOR [95 % CI]^e,f^	1.00	1.16 [0.75–1.81]	0.67	1.31 [0.79–2.18]	0.293
Body Mass Index^i^					
Healthy: 18.5–24.9 kg/m^2^	40.6 [39.1–42.1]	46.1 [37.2–55.2]		47.5 [35.8–59.5]	
**Underweight: <18.5 kg/m** ^**2**^	1.1 [0.9–1.5]	2.7 [1.3–5.3]		2.4 [0.9–6.1]	
**Overweight: 25–30 kg/m** ^**2**^	40.0 [38.4–41.6]	28.4 [21.1–36.9]		37.3 [26.9–49.0]	
Obese: >30 kg/m^2^	18.3 [16.5–20.3]	22.9 [13.5–38.2]		12.8 [4.6–29.6]	
aAOR [95 % CI]^e,f^	1.00	0.77 [0.54–1.11]	0.156	0.68 [0.44–1.07]	0.094
Screened positive for depression at interview^j^	8.9 [8.1–9.8]	8.9 [5.5–14.3]		6.4 [3.5–11.7]	
aAOR [95 % CI]^e^	1.00	1.03 [0.62–1.70]	0.909	0.92 [0.48–1.77]	0.797
Received treatment for depression from a health professional, past year	5.8 [5.1–6.5]	14.0 [9.2–20.5]		19.9 [11.9–31.4]	
aAOR [95 % CI]^e^	1.00	2.75 [1.69–4.47]	<0.001	4.03 [2.30–7.07]	<0.001
Any health condition/disability that has affected sexual activity/enjoyment, past year	15.3 [14.2–16.5]	27.1 [19.9–35.7]		29.3 [19.2–42.1]	
aAOR [95 % CI]^e^	1.00	2.24 [1.48–3.39]	<0.001	2.57 [1.52–4.37]	<0.001

All estimates are age-standardised

^*^ *p*-value for comparing all MSM to MSEW

^**^ *p*-value for comparing MSM who identified as gay in Natsal-3 to MSEW. Note that MSM who identify as gay are a subset of all MSM

^a^Unweighted, weighted denominators defined as participants who reported at least one sexual partner in the 5 years prior to interview

^b^MSM who identified as gay in Natsal-3 are a subset of all MSM

^c^Defined as usual alcohol consumption greater than recommended levels of 21 units per week [34]

^d^Defined as usually drinking more than 8 units of alcohol on one occasion [34]

^e^aAOR = age-adjusted odds ratio

^f^Age-adjusted odds ratio for reporting the responses in bold font [for those variables with >2 response options] relative to MSEW

^g^In contrast to reporting ‘very good’, ‘good’, or ‘fair’

^h^Measure of comorbidity includes arthritis, heart attack, coronary heart disease, angina, other forms of heart disease, hypertension, stroke, diabetes, broken hip or pelvis bone or hip replacement ever, backache lasting >3 months, any other muscle or bone disease lasting >3 months, cancer, and any thyroid condition treated in the past year [4]

^i^Body Mass Index thresholds as per National Obesity Observatory/Public Health England [35]

^j^As per PHQ-2 [15, 16]

### Sexual behaviours and sexual health outcomes

In addition to MSM being more likely than MSEW to report larger numbers of partners (Table 2), including reporting ten or more partners (aAOR:11.69, Table 5) and condomless sex with two or more partners (aAOR:3.52), both in the past year, they were also more likely to have had concurrent partners in the past five years (aAOR:5.65). However, there was no difference in the proportions reporting condomless *first* sex with their three most recent partners (past year). Almost one in five MSM reported taking drugs to assist sexual performance in contrast to 6.5 % of MSEW (past year, aAOR:3.50).

**Table 5:** Sexual behaviours, risk perception, and sexual health outcomes reported by men who have sex exclusively with women (MSEW), all men who have sex with men (MSM), and MSM who identified as gay in Natsal-3

	MSEW	All MSM		MSM who identified as gay^b^	
*Denominators:* ^*a*^	*5069, 6257*	*190, 191*		*107, 99*	
	% [95 % CI]	% [95 % CI]	*p*-value^*^	% [95 % CI]	*p*-value^**^
*Sexual behaviours*					
≥10 partners^c^, past year	1.2 [0.9–1.5]	13.4 [8.0–21.6]		19.6 [10.5–33.7]	
aAOR [95 % CI]^d^	1.00	11.69 [6.06–22.54]	<0.001	15.91 [7.10–35.66]	<0.001
Non-use of condoms at first sex with any of the three most recent partners^c^, past year	10.1 [9.3–11]	15.2 [9.7–23.1]		6.0 [3.2–10.9]	
aAOR [95 % CI]^d^	1.00	1.50 [0.91–2.49]	0.114	0.66 [0.35–1.24]	0.198
Condomless sex with ≥2 partners^c^, past year	8.1 [7.3–8.9]	24.6 [17.1–34.1]		13.0 [6.6–24.3]	
aAOR [95 % CI]^d^	1.00	3.52 [2.13–5.83]	<0.001	1.86 [0.85–4.07]	0.120
Concurrent partners^c^, past 5 years	15.6 [14.5–16.7]	52.4 [43.5–61.1]		41.6 [29.7–54.5]	
aAOR [95 % CI]^d^	1.00	5.65 [3.85–8.30]	<0.001	3.66 [2.23–6.00]	<0.001
Taken drugs to assist sexual performance, past year	6.5 [5.7–7.3]	19.1 [12.6–27.7]		18.4 [9.8–31.7]	
aAOR [95 % CI]^d^	1.00	3.50 [2.06–5.93]	<0.001	3.72 [1.74–7.94]	0.001
*STI/HIV risk perception*					
Self-perceived STI risk					
Not at all at risk	73.6 [72.2–74.9]	31.5 [23.7–40.7]		33.6 [22.4–46.9]	
Not very much	23 [21.7–24.3]	51.1 [42.3–59.8]		45.8 [34.1–58.0]	
**Quite a lot**	2.6 [2.2–3]	11.9 [7.1–19.5]		15.7 [8.6–27.0]	
**Greatly at risk**	0.9 [0.7–1.2]	5.4 [2.3–12.4]		4.9 [1.7–13.4]	
AOR [95 % CI]^e^	1.00	5.60 [3.28–9.56]	<0.001	7.04 [3.69–13.43]	<0.001
Self-perceived HIV risk					
Not at all at risk	76.0 [74.7–77.3]	35.0 [27.0–43.9]		39.2 [28.2–51.5]	
Not very much	21.0 [19.8–22.3]	50.6 [41.6–59.5]		44.5 [33.0–56.7]	
**Quite a lot**	1.9 [1.6–2.4]	9.5 [5.9–14.9]		10.9 [6.0–19.1]	
**Greatly at risk**	1.1 [0.8–1.4]	4.9 [2.1–11.2]		5.3 [2.0–13.7]	
AOR [95 % CI]^e^	1.00	5.44 [3.26–9.08]	<0.001	6.42 [3.67–11.23]	<0.001
*Sexual health outcomes*					
Sexual health clinic attendance, past year	14.1 [12.5–16]	33.0 [24.8–42.4]		31.9 [22.0–43.7]	
AOR [95 % CI]^e^	1.00	4.71 [2.58–8.58]	<0.001	4.53 [2.30–8.94]	<0.001
Tested for HIV, past year	3.4 [2.9–4]	17.0 [12.0–23.4]		21.7 [14.9–30.4]	
AOR [95 % CI]^e^	1.00	6.08 [3.92–9.41]	<0.001	9.44 [5.59–15.96]	<0.001
STI diagnosis, past year	0.9 [0.7–1.2]	4.9 [2.6–9.2]		6.3 [2.9–13.3]	
AOR [95 % CI]^e^	1.00	5.67 [2.67–12.04]	<0.001	7.85 [3.27–18.83]	<0.001
Lowest quintile of distribution of scores for Natsal-SF^f^	19.7 [18.4–21.1]	36.2 [28.2–45.2]		28.0 [17.9–40.9]	
AOR [95 % CI]^e^	1.00	2.28 [1.57–3.33]	<0.001	1.60 [0.95–2.69]	0.079
Feel distressed/worried about sex life	10.2 [9.3–11.2]	14.1 [9.0–21.2]		12.7 [6.3–24.1]	
AOR [95 % CI]^e^	1.00	1.39 [0.85–2.27]	0.194	1.14 [0.57–2.29]	0.703
Sought professional help/advice for sex life, past year	7.1 [6.3–7.9]	16.5 [11.0–24.0]		14.2 [8.1–23.7]	
AOR [95 % CI]^e^	1.00	2.70 [1.65–4.42]	<0.001	3.04 [1.62–5.68]	0.001
Experienced attempted non-volitional sex, ever	4.3 [3.7–5.0]	22.8 [16.0–31.4]		24.6 [15.1–37.4]	
AOR [95 % CI]^e^	1.00	6.51 [4.22–10.06]	<0.001	6.30 [3.66–10.83]	<0.001

All estimates are age-standardised

^*^ *p*-value for comparing all MSM to MSEW

^**^ *p*-value for comparing MSM who identified as gay in Natsal-3 to MSEW. Note that MSM who identify as gay are a subset of all MSM

^a^Unweighted, weighted denominators defined as participants who reported at least one sexual partner in the 5 years prior to interview

^b^MSM who identified as gay in Natsal-3 are a subset of all MSM

^c^Partners of either gender

^d^Age-adjusted odds ratio (aAOR) for reporting response relative to MSEW

^e^AOR - odds ratio for reporting response relative to MSEW adjusted for age, reporting recreational drug use, being treated depression (past year), educational attainment, smoking status, ethnicity, health condition that has affected sexual activity, and total partners numbers in the past 5 years

^f^Among men reporting sex in the past year [14]

MSM perceived greater STI/HIV risk than MSEW, although only a minority of MSM considered themselves ‘quite a lot’/‘greatly’ at risk of either STIs or HIV. Similarly, a minority of MSM reported sexual health clinic attendance (33.0 %), testing for HIV (17.0 %), and STI diagnosis/es (4.9 %), each in the past year, but these were all more commonly reported by MSM than MSEW, including after adjusting for key sociodemographics, health-related factors, and partner numbers (AORs: 4.71, 6.08, and 5.67, respectively).

While MSM were as likely as MSEW to report feeling distressed/worried about their sex lives, MSM were more likely than MSEW to report seeking professional help/advice for their sex life in the past year, and specifically from sexual health/GUM/STI clinics if they did (data not shown). Among sexually-active men, MSM were more likely than MSEW to have poorer overall sexual function according to the Natsal-SF [14] (AOR:2.28).

Among all MSM, 22.4 % reported having ever experienced attempted non-volitional sex, with the corresponding proportion estimate significantly smaller for MSEW at 4.3 % differences that remained in multivariable analyses (AOR:6.51).

### Clustering of harmful health behaviours and poor health outcomes

MSM were not only more likely than MSEW to report a number of harmful behaviours, but also to do so in combination *within* both the physical and sexual health domains (Table 6; corresponding aAORs:2.09 [95 % CI:1.55–2.82] and 3.40 [95 % CI:2.10–5.48], respectively). Furthermore, proportional Venn diagrams show that MSM were more likely than MSEW to report harmful behaviours *across* the physical health and sexual health domains, with 25.9 % of MSM in comparison to 10.4 % of MSEW reporting harmful behaviours in both domains.

**Table 6:** The extent that harmful behaviours^a^ cluster within and across health domains for (i) men reporting sex exclusively with women (MSEW), (ii) men reporting sex with men (MSM), and (iii) and MSM who identified as gay in Natsal-3

Population:	(i) Men who have sex exclusively with women (MSEW)		
Clustering:	*Within* domain		*Across* domains
Domain:	Physical health	Sexual health	
	% (95 % CI)	% (95 % CI)	
No. of harmful behaviours reported within each domain:			
0	54.5 (52.8–56.1)	86.3 (85.3–87.3)	
1	24.6 (23.3–26.1)	8.7 (7.9–9.6)	
2	14.8 (13.7–15.9)	4.7 (4.1–5.3)	
3	5.0 (4.4–5.7)	0.4 (0.2–0.5)	
4	1.2 (0.9–1.5)	N/A	
	*Total: 100 %*	*Total: 100 %*	
Population:	(ii) Men who have sex with men (MSM)		
Clustering:	*Within* domain		*Across* domains
Domain:	Physical health	Sexual health	
	% (95 % CI)	% (95 % CI)	
No. of harmful behaviours reported within each domain:			
0	34.5 (26.7–43.1)	64.2 (54.0–73.3)	
1	36.4 (28.0–45.7)	18.8 (12.3–27.5)	
2	16.4 (11.5–22.9)	15.6 (8.8–26.1)	
3	8.9 (4.9–15.7)	1.4 (0.4–4.9)	
4	3.8 (2.0–7.3)	N/A	
	*Total: 100 %*	*Total: 100 %*	
Population:	(iii) MSM who identified as gay^b^		
Clustering:	*Within* domain		*Across* domains
Domain:	Physical health	Sexual health	
	% (95 % CI)	% (95 % CI)	
No. of harmful behaviours reported within each domain:			
0	34.1 (24.7–44.9)	75.7 (61.7–85.7)	
1	40.9 (29.5–53.4)	12.7 (5.7–25.9)	
2	16.4 (10.0–25.8)	10.6 (4.3–23.9)	
3	4.0 (1.4–11.1)	1.1 (0.3–4.5)	
4	4.7 (2.2–9.9)	N/A	
	*Total: 100 %*	*Total: 100 %*	

^a^See Table 1

^b^MSM who identified as gay in Natsal-3 are a subset of all MSM

There was no difference in the number of poor physical health outcomes reported by MSM and MSEW (aAOR:1.02, 95 % CI:0.71–1.48, Table 7). In contrast MSM were more likely than MSEW to report one or more of the poor sexual health outcomes considered and to do so in combination within this domain (aAOR:3.42, 95 % CI:2.30–5.08). Furthermore, a larger proportion of MSM reported poor health outcomes across multiple domains (32.7 %, 95 % CI:24.8 %–41.7 %) relative to MSEW (15.0 %, 95 % CI:13.9 %–16.2 %), with 8.4 % of all MSM having experienced poor health outcomes in all three domains – physical, mental, and sexual health - in contrast to 1.5 % of all MSEW.

**Table 7:** The extent that poor health outcomes^a^ cluster within and across health domains for (i) men reporting sex exclusively with women (MSEW), (ii) men reporting sex with men (MSM), and (iii) and MSM who identified as gay in Natsal-3

Population:	(i) Men who have sex exclusively with women (MSEW)			
Clustering:	*Within* domain			*Across* domains
Domain:	Physical health	Sexual health	Mental health	
	% (95 % CI)	% (95 % CI)	% (95 % CI)	
No. of poor health outcomes reported within each domain:				
0	26.5 (25.2–27.9)	85.8 (84.7–86.9)	94.2 (93.5–94.9)	
1	40.4 (38.8–42.0)	13.3 (12.3–14.4)	5.8 (5.1–6.5)	
2	18.1 (16.8–19.4)	0.9 (0.6–1.2)	N/A	
3	13.1 (12.1–14.3)	0.0 (0.0–0.1)	N/A	
4	1.9 (1.5–2.4)	N/A	N/A	
	*Total: 100 %*	*Total: 100 %*	*Total: 100 %*	
Population:	(ii) Men who have sex with men (MSM)			
Clustering:	*Within* domain			*Across* domains
Domain:	Physical health	Sexual health	Mental health	
	% (95 % CI)	% (95 % CI)	% (95 % CI)	
No. of poor health outcomes reported within each domain:				
0	30.1 (22.8–38.6)	64.6 (55.6–72.7)	86.0 (79.5–90.8)	
1	33.5 (25.3–42.8)	28.7 (21.3–37.4)	14.0 (9.2–20.5)	
2	15.6 (10.0–23.4)	6.1 (2.8–13.0)	N/A	
3	17.8 (12.0–25.5)	0.6 (0.1–3.9)	N/A	
4	3.1 (1.2–8.0)	N/A	N/A	
	*Total: 100 %*	*Total: 100 %*	*Total: 100 %*	
Population:	(iii) MSM who identified as gay^b^			
Clustering:	*Within* domain			*Across* domains
Domain:	Physical health	Sexual health	Mental health	
	% (95 % CI)	% (95 % CI)	% (95 % CI)	
No. of poor health outcomes reported within each domain:				
0	31.4 (22.1–42.4)	61.1 (49.3–71.7)	80.1 (68.6–88.1)	
1	25.9 (17.1–37.2)	34.3 (24.4–45.7)	19.9 (11.9–31.4)	
2	11.1 (5.3–21.8)	4.6 (1.8–11.3)	N/A	
3	26.7 (17.1–39.1)	0.0 (N/A)	N/A	
4	4.9 (1.3–16.6)	N/A	N/A	
	*Total: 100 %*	*Total: 100 %*	*Total: 100 %*	

^a^See Table 1

^b^MSM who identified as gay in Natsal-3 are a subset of all MSM

### Summary comparing gay-identifying MSM to MSEW

When comparing *gay-identifying* MSM to MSEW, we observed similar patterns to those described above when comparing *all* MSM to MSEW, reflecting how gay-identifying MSM constitute half the sample of all MSM. Some differences exist though. Gay-identifying MSM were younger than MSEW, with half aged under 35, and so were also less likely to be in a steady or cohabiting relationship. Gay-identifying MSM were more likely than MSEW to live in urban areas, and often areas of greatest deprivation. The health behaviours of gay-identifying MSM differed to MSEW in similar ways to those observed for all MSM relative to MSEW, especially regarding greater reporting of recreational drug use. However, the prevalence of the adverse health *outcomes* studied was often higher for gay-identifying MSM relative to all MSM (although confidence intervals overlap), and thus aAORs were larger, e.g., health status reported as ‘bad’ or ‘very bad’ (aAORs 2.23 vs. 1.67, respectively, relative to MSEW). The largest difference in aAORs between gay-identifying MSM and all MSM, relative to MSEW, was observed for reporting receiving treatment for depression in the past year (aAORs 4.03 vs. 2.75, respectively, although confidence intervals again overlap). This suggestion of greater inequality for gay-identifying MSM was less evident in terms of sexual behaviours and outcomes. For example, the aAORs were smaller for gay-identifying MSM than for all MSM for reporting condomless sex and concurrency. For the sexual health outcomes studied, the differences with MSEW were similar, except that gay-identifying MSM were as likely as MSEW to have poor sexual function, while the aAOR for poor sexual function was significantly greater for *all* MSM relative to MSEW. In summary, and as evident from Tables 6 and 7, these findings suggest marginally greater clustering of poor health outcomes (but not poor health behaviours) for gay-identifying MSM than for all MSM, and in turn, for MSEW.

## Discussion

National probability survey data show that only a small minority of men in Britain are MSM in terms of reporting sex with men in the past five years, and of these men, around half identify as gay. While many of the harmful health behaviours and poor health outcomes studied were reported by a minority of MSM, this was often a larger proportion than observed among MSEW, and they were also more likely to be reported in combination and across health domains, demonstrating the considerable health inequality that exists for MSM in Britain, often regardless of whether or not MSM identify as gay.

A major strength of this study is that it uses data from a large national probability survey such that the findings can be considered as broadly representative of the British general population [11]. In addition, unlike the publication of data on MSM collected by Natsal-2 [19], this study has exploited the uniqueness of the Natsal data in terms of them being collected from all men, by considering the well-being of MSM relative to MSEW. The ability to sample from the large proportion of MSM who did not identify as gay is a further strength, and shows that a broader group of MSM are represented in this study than in previous studies, which have tended to sample from the gay scene in clubs or sexual health clinic attendees, and so have reported greater sexual risk and drug use behaviours and adverse sexual health outcomes [8-10, 20]. However, limitations are that the population prevalence of MSM results in a relatively small sample of MSM in Natsal, with insufficient statistical power to stratify MSM according to their sexual identity. While we presented estimates specifically for gay-identifying MSM alongside those for all MSM, the size of this sub-sample means that their estimates should be interpreted with caution. Including men who reported *ever* having had same-sex sex would have increased the number of MSM in our sample, but as some men may have had a transient experience we chose to focus on the past five years to minimise this effect, and also to present a contemporary picture of MSM in Britain. Some misclassification bias is also likely with our comparator group of MSEW as, by definition, this includes men who have not yet had, but who go on to have, male partners in the future, as well as MSEW who reported same-sex sex more than five years ago. However, the latter corresponds to a very small minority of MSEW (2.5 %), and so the in/exclusion of these 115 men has little impact on the magnitude of the estimates given the large number of MSEW in Natsal. Finally, as with all survey data we rely on what participants report so our data are subject to reporting and social desirability bias, and as a cross-sectional survey we are unable to assume causality.

An important strength of this paper is that it considers sexual health beyond STI/HIV transmission risk. By including a psychometrically-validated measure of sexual function, the Natsal-SF [14], and data about ever-experience of non-volitional sex, mental and physical health status and behaviours considered detrimental to health, we have been able to consider the wider health and well-being needs of MSM. Our endeavour is consistent with the rationale for the broader framing of sexual health [3], and Public Health England’s recent action plan for promoting the health and wellbeing of gay, bisexual and other MSM by taking into account their wider health issues and inequalities and the broader socio-economic and cultural context. [5] We recognise that the variables used to define the health domains we focused on are not exhaustive, reflecting the available data. Of note, Natsal-3 was relatively limited in capturing mental health, due to the complexity of doing so within a questionnaire with primarily a sexual health focus and the relatively low prevalence of conditions other than depression [4]. In this respect, we chose to focus on reported treatment for depression as we sought to identify individuals experiencing diagnosed depression at the more severe end of the clinical spectrum [4].

Natsal’s rich sexual behaviour data enabled us to examine the number of male *and* female sexual partners reported in various timeframes, including new partners and condomless sex partners. MSM reported larger numbers of partners on average compared to MSEW, as reported elsewhere [21]. However, the bimodal distribution of partner numbers we observed for MSM, with a small but non-negligible proportion of MSM reporting very few partners, challenges stereotypes inferred from clinical data and community surveys of gay men [8-10]. Additionally, considerably larger proportions of MSM reported female partners than observed in convenience surveys [9, 10], and as similar proportions of MSM and MSEW reported having new female partners in the past year, then our data support the notion that some MSM may act as a bridge population for STI/HIV transmission between higher-risk and lower-risk sexual networks [22].

Having larger numbers of sexual partners, and particularly multiple partners without using condoms, are both recognised as associated with a number of adverse sexual health outcomes, including STI/HIV acquisition [23], and both were more commonly reported by MSM than MSEW. Perhaps consequentially, MSM were more likely than MSEW to perceive themselves as at risk of STI/HIV acquisition, although the majority considered their risk as ‘not very much’. This may, in part, explain our finding that the majority of MSM reported that they had not been to a sexual health clinic and/or tested for HIV in the past year; figures that are far lower than observed from clinical audit [8, 24], suggested by convenience surveys [9, 10], or recommended by national guidelines [25, 26]. MSM were on the other hand more likely than MSEW to report seeking help/advice regarding their sex life (particularly from sexual health clinics) and to have taken drugs to assist their sexual performance. While this may suggest that MSM are better at seeking help for their sex lives than MSEW, it may reflect that MSM were more likely than MSEW to have poorer overall sexual function according to the validated Natsal-SF measure [14]. Although this hypothesis is consistent with findings from large surveys of gay men undertaken in the US and Australia that observed very high reported prevalence of sexual function problems [27, 28], it is worth noting that the difference in sexual function was smaller when the analysis was limited to gay-identifying MSM. Our finding of a higher prevalence of ever experiencing attempted non-volitional sex among MSM (regardless of whether they identified as gay) than among MSEW also supports other studies [29, 30].

As others have previously reported [6], we observed higher levels of poor mental health among MSM in contrast to MSEW, at least in terms of the proportion reporting receiving treatment for depression [31]. In addition, MSM reported greater substance use in terms of smoking and recreational drug use. However, to reiterate, many of the harmful health behaviours studied were often reported by only a minority of MSM, and considerably smaller proportions than observed from clinic and community surveys of MSM [8-10]. Furthermore, MSM and MSEW were often similar in terms of their physical health, contradicting previous research [32], although when we focused on gay-identifying MSM, these differences with MSEW were often larger. Health inequalities were also evident in the domain of sexual health with MSM more likely than MSEW to report both harmful sexual behaviours and poor sexual health outcomes, to report these in combination, and in conjunction with those in other health domains.

## Conclusions

These data suggest that there is a small but non-negligible group of MSM – and not necessarily MSM who identify as gay - who are likely to benefit from interventions that adopt a more holistic approach to improving health and well-being. However, these should not be at the expense of interventions that seek to tackle the ongoing STI/HIV epidemic among the MSM population [1, 2]. Policies and interventions that promote regular testing, condom use, and safe sexual behaviour among MSM at risk of infection [24-26] should therefore remain a priority in order to improve the health of individuals as well as public health. Achieving this in practice requires a two-pronged approach: Sexual health clinics need to offer MSM more holistic care, either in-house or through referral, while healthcare professionals in general medical services require a greater awareness of MSM (for example, by routinely asking about sexual orientation as the NHS recommends [33] as well as the gender of their sexual partners), and that their healthcare includes, but is not limited to the sexual health needs of MSM.

While this paper has sought to advance the literature by considering health inequalities beyond STIs and HIV, and has presented evidence of the extent to which health inequalities cluster for some MSM, future research needs to understand the mechanisms underlying these inequalities, how and why they cluster. Adopting an even broader perspective that goes beyond the proximal determinants of health, by seeking insight into distal determinants, for example in terms of the sociocultural context, will be helpful for gaining this understanding and for developing interventions that can effectively address multifaceted inequalities.

## Abbreviations

AOR, adjusted odds ratio; BMI, body mass index; CAPI, computer-assisted personal-interview; CASI, computer-assisted self-interview; HIV, human immunodeficiency virus; IMD, Index of Multiple Deprivation; MSEW, men who have with women; MSM, men who have sex with men; Natsal, National Survey of Sexual Attitudes and Lifestyles; NS-SEC, National Statistics Socio-Economic Classification; STI, sexually transmitted infection

## References

[1] Yin Z, Brown AE, Hughes G, Nardone A, Gill ON, Delpech V (2014). HIV in the United Kingdom 2014 Report: Data to end 2013.

[2] Public Health England (2015). Sexually transmitted infections and chlamydia screening in England, 2013. HPR.

[3] WHO. Defining sexual health: report of a technical consultation on sexual health, 28–31 January 2002, Geneva. 2006. http://www.who.int/reproductivehealth/publications/sexual_health/defining_sh/en/. (Accessed 26 Nov 2015).

[4] Field N, Mercer CH, Sonnenberg P (2013). Associations between health and sexual lifestyles in Britain: findings from the third National Survey of Sexual Attitudes and Lifestyles (Natsal-3). Lancet.

[5] Public Health England. Promoting the health and wellbeing of gay, bisexual and other men who have sex with men. London. 2014. https://www.gov.uk/government/publications/promoting-the-health-and-wellbeing-of-gay-bisexual-and-other-men-who-have-sex-with-men. (Accessed 26 Nov 2015).

[6] King M, Semlyen J, See Tai S (2008). A systematic review of mental disorder, suicide and deliberate self harm in lesbian, gay and bisexual people. BMC Psychiatry.

[7] Vosburgh HW, Mansergh G, Sullivan PS, Purcell DW (2012). A review of the literature on event-level substance use and sexual risk behavior among men who have sex with men. AIDS Behavior.

[8] Desai M, Desai S, Sullivan AK (2013). Audit of HIV testing frequency and behavioural interventions for men who have sex with men: policy and practice in sexual health clinics in England. Sex Transm Infect.

[9] Reid D (2011). Vital Statistics 2010: The UK Gay Men’s Sex Survey Data Report: All England by Strategic Health Authority of Residence.

[10] Schwarcz S, Spindler H, Scheer S, Valleroy L, Lansky A (2007). Assessing representativeness of sampling methods for reaching men who have sex with men: a direct comparison of results obtained from convenience and probability samples. AIDS Behav.

[11] Erens B, Phelps A, Clifton S (2013). Methodology of the third British National Survey of Sexual Attitudes and Lifestyles (Natsal-3). Sex Transm Infect.

[12] Johnson AM, Mercer CH, Erens B (2001). Sexual behaviour in Britain: partnerships, practices, and HIV risk behaviours. Lancet.

[13] Ruf M, Delpech V, Osuagwu U, Brown AE, Robinson E, Chadborn T (2011). Men who have sex with men: estimating the size of at-risk populations in London primary care trusts. Int J STD AIDS.

[14] Mitchell KR, Ploubidis GB, Datta J, Wellings K (2012). The Natsal-SF: a validated measure of sexual function for use in community surveys. Eur J Epidemiol.

[15] Arroll B (2003). Screening for depression in primary care with two verbally asked questions: cross sectional study. BMJ.

[16] Arroll B, Goodyear-Smith F, Crengle S (2010). Validation of PHQ-2 and PHQ-9 to screen for major depression in the primary care population. Ann Fam Med.

[17] Office for National Statistics (2010). Standard Occupational Classification 2010.

[18] Payne RA, Abel GA (2012). UK indices of multiple deprivation—a way to make comparisons across constituent countries easier. Health Stat Q.

[19] Mercer CH, Fenton KA, Copas AJ (2004). Increasing prevalence of male homosexual partnerships and practices in Britain 1990–2000: Evidence from national probability surveys. AIDS.

[20] Prah P, Hickson F, Bonell C (2016). Men who have sex with men in Great Britain: comparing methods and estimates from probability and convenience sample surveys. Sex Transm Infect.

[21] Centers for Disease Control and Prevention. HIV risk, prevention, and testing behaviors—National HIV behavioral surveillance system: Men who have sex with men, 20 U.S. cities, 2011. HIV Surveillance Special Report 8. 2014. http://www.cdc.gov/hiv/library/reports/surveillance/#special. (Accessed 20 Apr 2016).

[22] Gorbach PM, Murphy R, Weiss RE, Hucks-Oritz C, Shoptaw S (2009). Bridging sexual boundaries: Men who have sex with men and women in a street-based sample in Los Angeles. J Urban Health.

[23] Sonnenberg P, Clifton S, Beddows S (2013). Prevalence, risk factors, and uptake of interventions for sexually transmitted infections in Britain: findings from the National Surveys of Sexual Attitudes and Lifestyles (Natsal). Lancet.

[24] McClean H, Sullivan AK, Carne CA (2012). UK national audit against the key performance indicators in the British Association for Sexual Health and HIV Medical Foundation for AIDS and Sexual Health Sexually Transmitted Infections Management Standards. Int J STD AIDS.

[25] National Institute for Health and Clinical Excellence (2011). Increasing the Uptake of HIV Testing Among men who Have sex With men.

[26] BASHH (2014). Recommendations for Testing for Sexually Transmitted Infections in men who Have sex With men.

[27] Hirshfield S, Chiasson MA, Wagmiller RL (2010). Sexual dysfunction in an Internet sample of U.S. men who have sex with men. J Sex Med.

[28] Mao L, Newman CE, Kidd MR, Saltman DC, Rogers GD, Kippax SC (2009). Self-reported sexual difficulties and their association with depression and other factors among gay men attending high HIV-caseload general practices in Australia. J Sex Med.

[29] Coxell A, King M, Mezey G, Gordon D (1999). Lifetime prevalence, characteristics, and associated problems of non-consensual sex in men: cross sectional survey. BMJ.

[30] Walters M, Chen J, Breiding M (2013). The National Intimate Partner and Sexual Violence Survey (NISVS): 2010 Findings on Victimization by Sexual Orientation.

[31] Field N, Prah P, Mercer CH (2016). Are depression and poor sexual health neglected co-morbidities? Evidence from a population sample. BMJ Open.

[32] Lick DJ, Durson LE, Johnson KL (2013). Minority stress and physical health among sexual minorities. Perspect Psychol Sci.

[33] NHS England/Equality and Health Inequalities Team. Monitoring equalities and health inequalities: A position paper. 2015. https://www.england.nhs.uk/wp-content/uploads/2015/03/monitrg-ehi-pos-paper.pdf. (Accessed 26 Nov 2015).

[34] NHS Choices website. Binge drinking. The Information Standard member organisation. http://www.nhs.uk/Livewell/alcohol/Pages/Bingedrinking.aspx. (Accessed 20 Apr 2016).

[35] National Obesity Observatory (2009). Body Mass Index as a Measure of Obesity.

